# Pay-off-biased social learning underlies the diffusion of novel extractive foraging traditions in a wild primate

**DOI:** 10.1098/rspb.2017.0358

**Published:** 2017-06-07

**Authors:** Brendan J. Barrett, Richard L. McElreath, Susan E. Perry

**Affiliations:** 1 Animal Behavior Graduate Group, University of California, Davis, CA, USA; 2 Department of Anthropology, University of California, Davis, CA, USA; 3 Department of Human Behavior, Ecology, and Culture, Max Planck Institute for Evolutionary Anthropology, Leipzing, Germany; 4 Department of Anthropology, University of California, Los Angeles, CA, USA; 5 Center for Behavior, Evolution, and Culture, University of California, Los Angeles, CA, USA

**Keywords:** pay-off bias, social learning, behavioural traditions, *Cebus*, cultural transmission, extractive foraging

## Abstract

The type and variety of learning strategies used by individuals to acquire behaviours in the wild are poorly understood, despite the presence of behavioural traditions in diverse taxa. Social learning strategies such as conformity can be broadly adaptive, but may also retard the spread of adaptive innovations. Strategies like pay-off-biased learning, by contrast, are effective at diffusing new behaviour but may perform poorly when adaptive behaviour is common. We present a field experiment in a wild primate, *Cebus capucinus*, that introduced a novel food item and documented the innovation and diffusion of successful extraction techniques. We develop a multilevel, Bayesian statistical analysis that allows us to quantify individual-level evidence for different social and individual learning strategies. We find that pay-off-biased and age-biased social learning are primarily responsible for the diffusion of new techniques. We find no evidence of conformity; instead rare techniques receive slightly increased attention. We also find substantial and important variation in individual learning strategies that is patterned by age, with younger individuals being more influenced by both social information and their own individual experience. The aggregate cultural dynamics in turn depend upon the variation in learning strategies and the age structure of the wild population.

## 1. Introduction

The existence of culture or behavioural traditions [1] in non-human animals has been a topic of intrigue to evolutionary biologists and ethologists for centuries [2–4]. Recently, research interest in animal cultures has soared, partially driven by findings from long-term cross-site collaborations within primatology [5–7] and cetaceology [8,9] in the early twenty-first century. As the diversity of taxa in which social learning is studied grows, it appears that traditions might be more widespread and ecologically meaningful than was previously appreciated.

As evidence accumulates, the study of cultural mechanisms has shifted focus from asking ‘can animals learn socially?’ to ‘how and under what conditions do animals learn socially?’. The ecological drivers that favour social learning are theoretically well explored [10]. The mechanistic details and evolutionary and ecological consequences of social learning are less well understood. From an individual's perspective, it may be difficult to know whom or exactly what to copy. To cope with these difficulties, organisms use heuristics and strategies [10–12] to minimize the costs and increase the efficiency of social learning. Variation in learning strategy, whether between individuals or over the life course, may also be important [13–15].

Different strategies have different advantages. Two families of social learning strategies that have received both theoretical and empirical attention are conformity and pay-off bias [10,16,17]. Conformist transmission, or positive frequency dependence, can be adaptive especially in spatially heterogeneous environments [10,18,19]. However, unless it is combined with other, flexible strategies, conformity may prohibit more adaptive behaviours from spreading [18,20] or cause population collapse [21]. In contrast with conformity, pay-off-biased social learning is very effective at spreading novel adaptations. Pay-off-biased social learning attends to behaviour that is associated with higher pay-offs and presumably increased fitness. However, it can be outperformed by conformity, once adaptive behaviour is common [22].

There is empirical evidence for both conformist and pay-off-biased social learning in humans [17]. In other animals, conformity [23,24] has been studied more extensively than pay-off bias. To our knowledge, no non-human study has directly compared the explanatory power of conformity and pay-off-biased social learning.

Here, we report results from a field experiment with white-faced capuchin monkeys (*Cebus capucinus*) that is capable of distinguishing conformist and pay-off-biased social learning. Capuchins are an excellent study system for understanding social learning and traditions. They are tolerant of foraging in proximity with conspecifics [25], independently evolved many brain correlates associated with intelligence [26,27], and display the largest recorded repertoire of candidate behavioural traditions of any platyrrhine: social conventions [7], interspecific interactions [28] and extractive foraging techniques [29–32]. Their reliance on social learning, frequency of innovation and complexity of social interactions exemplifies what is predicted for long-lived animals with a slow life-history strategy [33]. We investigated the innovation and transmission of extractive foraging techniques used to access the protected seeds of the *Sterculia apetala* fruit. This fruit occurs sporadically over the range of *C. capucinus*. Only some groups are experienced with it. By introducing the fruit to a naive group in controlled settings, we observed the rise and spread of new foraging traditions. We then inferred which social learning strategies best predict individual behaviour and how they influence the origins and maintenance of traditions.

The statistical analysis employs a multilevel (aka hierarchical or varying effects) dynamic learning model, of the form developed by McElreath *et al.* [17], and inference is based upon samples from the full posterior distribution, using Hamiltonian Monte Carlo [34]. This model allows estimation of unique social and individual learning strategies for each individual in the sample. The analysis utilizes dynamic social network data, which were available during each field experimental session. It also permits examination of the relationship between any individual state (i.e. age, rank) and learning strategy. The multilevel approach makes it possible to apply these models to field data that lack precise balance and repeatedly sample individuals. We provide all code needed to replicate our results and to apply this same approach to any group time series of behaviour.

We document that the capuchins innovated a number of successful techniques. However, these techniques vary in their physical and time requirements. The statistical analysis suggests that pay-off-biased social learning was responsible for this spread of the quickest, most successful techniques through the group. We find no evidence of conformity, but do find evidence of weak anti-conformity—rare techniques attracted more attention. We also find evidence of an age bias in social learning, in which older individuals were more likely to be copied. Individuals varied in how they made use of social cues and individual experience, and age was a strong predictor. Our results comprise the first application of multilevel, dynamic social learning models to a study of wild primates and suggest that pay-offs to behaviour can have important and different influences on social and individual learning. Methodologically, the approach we have developed is flexible and practical, and allows for a stronger connection between theoretical models of learning and the statistical models used to analyse data.

## 2. Study design

### (a) Study system

This study was conducted between 2013 and 2015 on a group of habituated white-faced capuchin monkeys in and near Reserva Biológica Lomas Barbudal (RBLB) in northwest Costa Rica, during the months of December–February (see the electronic supplemental material and [35,36] for additional information about field site).

Capuchins heavily rely on extractive foraging to exploit difficult-to-access resources; this makes them an excellent comparative study system for understanding the evolution of extractive foraging in humans [26]. In neotropical dry forests, capuchins increase their reliance on extractive foraging during seasonal transitions when resources are limited. Capuchins receive more close, directed attention from conspecifics when they are foraging on large, structurally protected foods [37]. Many of the techniques required to access protected foods are candidate behavioural traditions [29].

Panamá fruits, *S. apetala*, are a dietary staple of capuchins at RBLB; they comprise 8% of the diet of most groups in the early dry season [37]. The fruits are *empanada*-shaped, and the fatty, protein rich seeds within are protected by a hardened outer husk and stinging hairs [38]. Instead of waiting for fruits to dehisce, capuchins will open closed fruits and work around their structural defences, thus reducing competition with other organisms. Panamá fruits require multiple steps to effectively open, process and consume, and panamá foraging generates the second highest level of close-range observation from conspecifics at RBLB [37]. Panamá processing techniques are also observed to vary between groups at RBLB and other field sites in the area [29], suggesting they are socially learned traditions. Wild capuchins without prior exposure to panamá fruits cannot initially open them [38], suggesting that personal experience and/or social influence are important.

Panamá processing techniques differ in efficiency, measured by the average time it takes to open a fruit. Techniques also differ in efficacy, both in their probability of being successful and due to costs incurred by encountering stinging hairs. This contrasts with other extractive foraging traditions that show no difference in efficiency or efficacy [30].

The focal group of this study, Flakes group (*n* = 25), fissioned from the original study group in 2003. They migrated to a previously unoccupied patch of secondary agricultural and cattle-ranching land characterized by riparian forest, pasture and neotropical oak woodland, where panamá trees are almost non-existent as they typically grow in evergreen, primary forests. Group scan data collected on foraging capuchins at RBLB from 2003 to 2011 show that Flakes was never observed foraging panamá, whereas other groups spent up to 1.21% of their annual foraging time eating panamá (electronic supplementary material, table S1). Two trees were found in the territory during phenological surveys, but are at the periphery, have small crowns and are in areas of the habitat shared with other capuchin groups. When this study was designed, veterans of the field site had no recollection of observing Flakes foraging for panamá. Observations of two natal Flakes adult males (old enough to be expert panamá foragers in any other group) found outside of their territory migrating suggest that they had little or no experience with panamá fruits.

Five adults in the group (two females and three males) grew up in different natal groups whose territories contained large numbers of panamá trees and whose groups exhibited higher rates of panamá foraging. For two migrant males from non-study groups, it is unknown if they previously learned to process panamá, but this seems likely, as evidenced by their skill. These individuals acted as models for different behaviours, as they differed in the primary panamá processing techniques they presumably acquired in their natal groups. By providing panamá fruits to both naive/inexperienced juveniles and to knowledgeable adult demonstrators who differ in processing techniques, we collected fine-grained data showing how inexperienced capuchins learn a natural behaviour.

### (b) Data collection

We collected panamá fruits from areas near RBLB for our experiment. Fruits were placed on a 25 cm-diameter wooden platform which provided visual contrast of the fruits against the ground as fruits blended with the leaf litter, and so the capuchins had some sort of naturalistic spatial cue to associate with panamá fruits. Two fruits were placed on 1–2 platforms in each experimental bout. This permitted 1–4 capuchins to forage at a given time, and two fruits per platform was the maximum number on which a single human observer could reliably collect data.

We placed multiple fruits for two reasons. First, when individuals are naturally foraging for panamá, they choose from multiple available fruits in a tree. Second, we wanted to see whom they bias their attention towards when given a choice of multiple potential demonstrators. While many learning experiments have one potential demonstrator to learn from in a foraging bout or assume that everyone observes that demonstrator, we believe that allowing them to choose a potential learning model is more representative of how wild animals learn.

Fruits were placed on platforms under a poncho to obscure the monkey's view of us handling fruits. As ponchos were worn regularly when not experimenting, monkeys were unlikely to associate their presence with panamá platforms. When monkeys were not looking, we uncovered the fruits and walked to an observation area away from the platform so that the monkeys could forage unimpeded. On digital audio recorders, we recorded if or when individuals saw, handled, processed, opened, ingested seeds from and dropped each fruit. We verbally described how they were processing each fruit (table 1) using an ethogram of techniques and which audience members observed them. Further information about fruit collection, data collection and observer training can be found in the electronic supplemental material text and video, in addition to video of panamá processing techniques.

**Table 1.** Summary statistics for the seven panamà processing techniques observed in this study. Mean and median duration presented in seconds.

technique	description	mean	median	% open	*n*
back attack	peel fibres off back from fruit with seam facing away from mouth, bite to pop open at seam	169.0	119	51.1	176
bite and pop	bite opposite corners of each fruit forcefully, bite to pop open at seam	49.7	29	37.8	283
canine seam	hold fruit perpendicular to mouth, insert upper and lower canines into seam to split open	70.5	42	88.5	511
chew hole	chew hole or rip fibres off fruit at corner, back, or side, seam not chewed	330.5	211.5	65.5	247
pound	pound fruit on hard substrate	n.a.	n.a.	0	15
scrub	scrub fruit on hard substrate	n.a.	n.a.	0	5
seam Strip	hold fruit parallel to mouth, strip fibres off along the seam, bite to pop open at seam	130.6	211.5	65.0	200
all techniques		131.5	95.0	65.6	1437

## 3. Statistical analyses

We analysed these data using multilevel experience-weighted attraction (EWA) models [39,40]. EWA models are a family of models that link individual learning rules and social information use to population-level dynamics by fitting existing mathematical models of learning as statistical models [16,17,41].

### (a) Social learning strategies

Our main focus is the contrast between two well-studied types of social learning: conformity and pay-off bias. However, we also investigate other plausible strategies. We quickly describe the background of these strategies and how the modelling framework incorporates them.

#### (i) Pay-off-biased learning

Copying the behaviour with the highest observable pay-off is a useful social learning strategy [22,42]. In a foraging context, selectively copying rate-maximizing behaviour can increase the efficiency of diet and resource acquisition. Guppies choose food patches with higher return rates [43], while wild tufted capuchins bias their attention towards the most efficient tool users [44]. Cues of pay-off may be noisy, however, and different individuals may require different techniques.

#### (ii) Model-biased learning

Sometimes evaluating the content of a behaviour is costly or impossible. In these circumstances, it may be an adaptive heuristic to bias attention towards particular demonstrators or ‘models’, who display cues (i.e. rank, health, fertility) that are likely to be correlated with adaptive behaviour.

Prestige-biased learning is a popular example of model bias in humans [45]. While animals may lack the concept of prestige, they have analogues. Captive chimpanzees have been found to be more likely to copy dominant individuals [41,46], while vervets copy same-sex high-ranking individuals [47].

Copying the behaviour of one's parents is another option. If a parent can survive and successfully reproduce, its offspring's existence serves as a cue that her parents are successful [48]. *Luehea* processing techniques of capuchins at RBLB were predicted by both the technique their mother used and the technique they saw performed most often [30]. Kin-biased learning has been found in many carnivores [49–51], but it is unclear whether this is due to cognition or is a consequence of family-unit social systems.

Copying similar individuals can be adaptive. Where individuals differ in strength, size or cognitive ability, it might be beneficial for learners to copy those who are most similar to them. Sex-biased learning has been found in several primate species [30,47].

#### (iii) Frequency-dependent learning

Frequency-dependent social learning occurs when frequency among demonstrators or frequency of demonstration influences adoption. It includes negative and positive frequency dependence. Negative frequency dependence, or anti-conformity, is preferentially copying rare behaviour. It may be a form of neophilia. Positive frequency dependence, known also as conformity or majority-rule, is preferentially copying the most common behaviour. Conformity can lead to the fixation and maintain the stability of a cultural trait [10,18]. Experiments in many captive [20,52–55] and some wild [23,24] animals have found evidence of conformist learning.

### (b) Model design

An EWA model comprises two parts: a set of expressions that specify how individuals accumulate experience and a second set of expressions that specify the probability of each option being chosen. Accumulated experience is represented by *attraction* scores, *A*_*ij*,*t*_, unique to each behaviour *i*, individual *j* and time *t*. A common formulation is to update *A*_*ij*,*t*_ with an observed pay-off *π*_*ij*,*t*_3.1
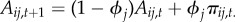
The parameter *ϕ*_*j*_ controls the importance of recent pay-offs in influencing attraction scores. This parameter is unique to individual *j*, and so can vary by age or any other feature.

To turn these attraction scores into behavioural choice, some function that defines a probability for each possible choice is needed. The conventional choice is a standard multinomial logistic, or *soft-max*, choice rule3.2
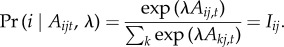
The parameter λ controls how strongly differences in attraction influence choice. When λ is very large, the choice with the largest attraction score is nearly always selected. When λ = 0, choice is random with respect to the attraction score. Individuals were assigned a pay-off of zero, *π*_*ij*,*t*_ = 0, if they failed to open a panamá fruit. If they were successful, pay-off was the inverse-log amount of time it took to open the fruit, *π*_*ij*,*t*_ = log(*T*_open_)^−1^. For the observed times *T*_open_, this ensures that pay-offs decline as *T*_open_ increases, but with the steepest declines early on.

Following previous work, social learning may influence choice directly and distinctly from individual learning. Let *S*_*ij*_ = *S*(*i*|***Θ***_*j*_) be the probability an individual *j* chooses behaviour *i* on the basis of a set of social cues and parameters ***Θ***_*j*_. Realized choice is given by3.3
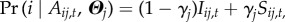
where *γ*_*j*_ is the weight, between 0 and 1, assigned to social cues. Under this formulation, social cues influence choice directly; attraction scores are influenced indirectly via the pay-offs associated with each individual's behavioural choice.

We incorporate social cues into the term *S*_*ij*,*t*_ by use of a multinomial probability expression with a log-linear component *B*_*ij*,*t*_ that is an additive combination of cue frequencies. Specifically, the probability of each option *i*, as a function only of social cues, is3.4
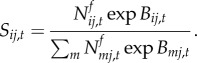
This is easiest to understand in pieces. The *N*_*ij*,*t*_ variables are the observed frequencies of each technique *i* at time *t* by individual *j*. The exponent *f* controls the amount and type of frequency dependence. When *f* = 1, social learning is unbiased by frequency and techniques influence choice in proportion to their occurrence. When *f* > 1, social learning is conformist. Other social cues, like pay-off, are incorporated via the *B*_*ij*,*t*_ term3.5
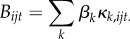
This is the sum of the products of the influence parameters *β*_*k*_ and the cue values *κ*_*k*,*ijt*_. We consider five cues:

(1)Pay-off. *κ* = log(*t*_open_)^−1^ or, for failure, *κ* = 0.
(2) Demonstrator rank. *κ* = 1 for alpha rank, 0 otherwise.
(3) Matrilineal kinship. *κ* = 1 for matrilineal kin, 0 otherwise.
(4) Age similarity. *κ* is defined as the inverse absolute age difference: (1 + |age_demonstrator_ − age_observer_|)^−1^.
(5) Age bias. *κ* = age_demonstrator_.

The final components needed are a way to make the individual-level parameters depend upon individual state and a way to define the window of attention for social cues at each time *t*. The parameters *γ*_*j*_ and *ϕ*_*j*_ control an individual *j*'s use of social cues and rate of attraction updating, respectively. We model these parameters as logistic transforms of a linear combination of predictors. For example, the rate of updating *ϕ*_*j*_ for an individual *j* is defined as follows:3.6
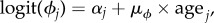
where *α*_*j*_ is a varying intercept per individual and *μ*_*ϕ*_ is the average influence of age on the log-odds of the updating rate. Social information available at each time step in the model was a moving window of the previous 14 days of observed foraging bouts. This allows new social information to be used, while old information is discarded. We tested the sensitivity of the time window used to calculate social cues and found our results were robust to variations in window width (7, 14, 21, 28 days) (electronic supplementary material, table S3). Attempts to parametrize window width fitted poorly. To fitted the model, we defined a global model incorporating all cues, using both parameter regularization and model comparison with sub-models to account for overfitting. Overall nine models were fitted, representing nine learning strategies (electronic supplementary material, table S2). Models were fitted using the Hamiltonian Monte Carlo engine Stan v. 2.14.1 [34], in R v. 3.3.2 [56]. We compared models using WAIC [57]. To check our approach, we simulated the hypothesized data generating process and pay-off structure and recovered data-generating values from our simulated data. We chose conservative, weakly informative priors for our estimated parameters. This made our models sceptical of large effects and helped ensure convergence.

## 4. Results: innovation and diffusion of techniques

Of the 25 individuals in the group, 23 tried to process panamá and 21 were successful at least once over 75 experimental days. We observed seven types of predominant fruit processing techniques on 1441 fruits, which varied in time required and the proportion of successful attempts (table 1). Mean (median) duration ranged from 50 (29) s to 330 (210) s. Proportion of successful attempts ranged from 0.38 to 0.89 (table 1).

**Figure 1.**
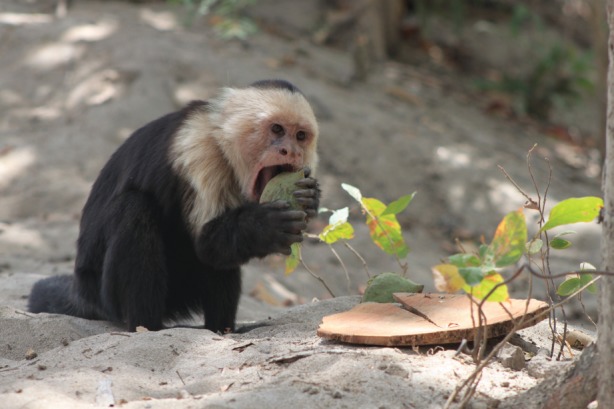
Adult male NP exhibits the canine seam technique. (Online version in colour.)

**Figure 2.**
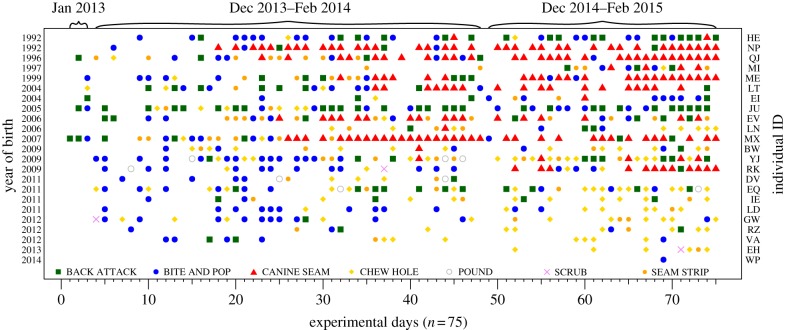
Techniques observed during experiment. Rows are unique individuals, from the oldest (top) to the youngest (bottom). The *x*-axis represents the sequential order of experimental days. Each colour/shape represents most common technique used by an individual on that day; no point indicates days of no processing. The most successful technique indicated by red triangles (canine seam) diffused to older members of the population. Younger individuals did not use canine seam.

The technique frequencies changed over time, in the group and in most individuals (figure 2; electronic supplementary material, figures S3 and S4). The most efficient technique, canine seam, went from non-existent in the group to the most common technique. It was introduced by an immigrant adult male (NP). Two knowledgeable adults, an adult female (ME) and the alpha male (QJ), switched to the canine seam technique. All others born after 2009 tried it at least once (electronic supplementary material, figure S4). However, canine seam never reached fixation in the population.

### (a) Results of experience-weighted attraction models

There was overwhelming support for some mix of individual and social learning over individual learning alone (electronic supplementary material, table S2). The highest-ranked model was the global model containing all strategies and age effects on learning parameters, which received 94% of the total model weight. We focus on this model, as it is both highest ranking and its parameter values agree with the weights assigned in the overall model set.

Marginal posterior distributions of each parameter are displayed in table 2 and visualized in electronic supplementary material, figure S1. Note that the marginal posterior distribution of each parameter cannot be directly interpreted as the importance of each factor in the total diffusion of behaviour. The weight of social information (*γ*), for example, can be relatively small at each instantaneous choice but still be decisive in determining which behaviour spreads, because individual discovery rates may be even smaller. As each individual's behaviour is unique to their observed social information, personal experience and estimated individual-level parameters, we encourage readers to view marginal predictions with visualizations of implied individual behaviour, using posterior predictive distributions in electronic supplementary material, figure S3.

**Table 2.** Posterior medians and standard deviations from the global model. Estimates of *σ*_individual_ are the standard deviations of varying effects for that parameter across individuals. Posteriors visualized in electronic supplementary material, figures S1 and S2.

parameter	λ	*ϕ*	*γ*	*f*	*β*_pay_	*β*_kin_	*β*_rank_	*β*_coho_	*β*_age_	*μ*_*ϕ*_	*μ*_*γ*_
posterior median	20.97	0.15	0.14	0.38	1.02	0.19	−0.11	0.48	0.69	−0.11	−0.10
posterior s.d.	1.11	0.03	0.03	0.28	0.84	0.93	0.91	0.93	0.92	0.03	0.05
*σ*_individual_	—	0.66	0.69	1.29	0.28	0.25	0.26	0.26	0.25	—	—

#### (i) Influence of conformity and pay-off bias (*f* and *β*_pay_)

**Figure 3.**
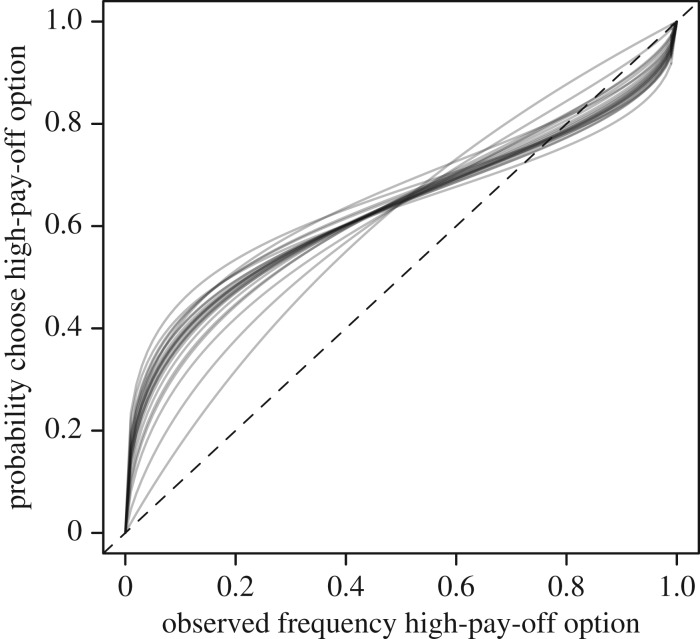
Posterior predictions of probabilities of choosing a socially observed option with pay-off log(*t*_open_)^−1^ = 0.5, relative to an observed option that was not successfully opened.

The raw marginal conformist exponent is below 1 on average, indicating mild anti-conformity—a bias towards copying rare behaviours. The marginal pay-off-bias coefficient is strongly positive, indicating attraction to high-pay-off actions. Figure 3 visualizes the individual social learning function *S*_*ijt*_ (expression (3.4)) implied when only conformity and pay-off bias are present. The horizontal axis is the observed frequency of a higher pay-off option among demonstrators. The vertical axis is the probability an individual chooses the higher pay-off option. Each curve in the figure represents the posterior mean for an individual. The diagonal dashed line represents unbiased social learning. All individuals are strongly biased by pay-off, resulting in a preference for the high-pay-off option over most of the range of the horizontal axis. But most individuals also display weak anti-conformity, resulting in a preference for the rarer, low-pay-off option in the upper right corner.

#### (ii) Weight of past experience (*ϕ*)

**Figure 4.**
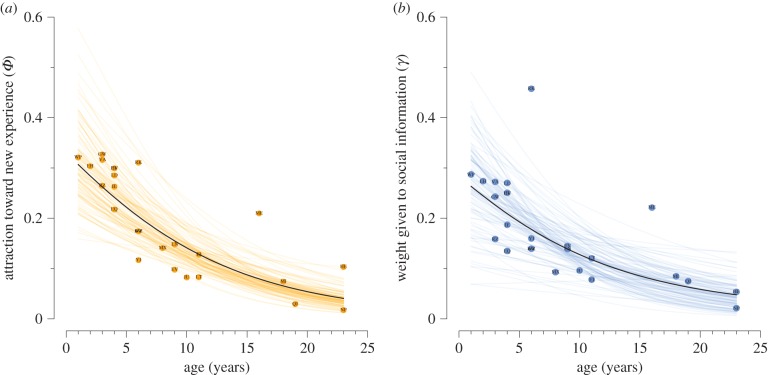
Relationships between age and (*a*) attraction to new experience (*ϕ*) and (*b*) influence of social information (*γ*). Black line represents the posterior mean. Solid points are posterior means of individual varying effects. Lighter lines are 100 posterior samples. (Online version in colour.)

On average, capuchins more heavily favour previous experiences over new ones (*ϕ* = 0.15; [0.11, 0.20] 89% credible interval), table 2). However, there is considerable individual variation in attraction to new experience (*σ*_individual_ = 0.66), ranging from 0.08 to 0.36, which was negatively predicted by age (*μ*_age_ = −0.11; 89% CI [−0.16, −0.06]; figure 4*a*). This suggests that older individuals are more canalized than younger individuals.

#### (iii) Weight of social information (*γ*)

*γ* estimates for individuals varied considerably, in the range of 0.07–0.39 (*σ*_individual_ = 0.66). *γ* was also negatively related to age (*μ*_age_ = −0.10; 89% CI [−0.18, −0.03]; figure 4*b*). This suggests that younger individuals rely more on social cues.

#### (iv) Age bias (*β*_age_)

Age bias contributed notably to social learning in our global model (*β*_age_ = 0.69; 89% CI [−0.79, 2.14]; table 2), suggesting that all capuchins were more likely to copy older demonstrators.

#### (v) Age similarity, kin and rank biases

None of age similarity, matrilineal kin or rank biases presented a strong or consistent effect (coho, kin and rank in table 2). While these strategies may have influenced some individuals and decisions, there is little evidence of general importance for these cues.

## 5. Discussion

We set out to examine the roles of conformist and pay-off-biased social learning among wild capuchin monkeys during the diffusion of novel food processing techniques. We find no evidence of conformity, defined as positive frequency dependence. We do, however, find strong evidence of pay-off-biased learning.

Little work has examined whether animals use pay-off-biased social learning. We do not know how common such strategies are in nature. It is common to experimentally examine pay-off-equivalent options, shedding no light on pay-off bias. The common exclusion approach to identifying animal culture accidentally excludes pay-off bias, by diagnosing ecologically correlated behavioural differences as non-cultural [5]. This may result in overlooking adaptive socially learned behaviour. If pay-off bias is common, this makes the problem of identifying animal traditions more subtle.

We also found evidence that other social cues, such as age, influence social learning. Age also modulated underlying learning parameters. In combination, these influences are sufficient to describe the diffusion and retention of successful foraging techniques within the group. In the remainder of the discussion, we elaborate on the findings and summarize some of the advantages and disadvantages of our approach.

### (a) Wild capuchins acquire extractive foraging techniques quickly via social learning

This study shows that one group of wild capuchin monkeys socially learn extractive foraging techniques from conspecifics and supports claims that food processing techniques are socially learned traditions. It has been challenging to find experimental evidence for social learning of object manipulation tasks in captive capuchins [26,58]. Better evidence for social learning might be found across a broader range of taxa if more ecologically valid behaviours are studied in the wild. This study also demonstrates that capuchins, like other animals [59], may be able to acquire new, efficient behaviour in a matter of days or weeks if knowledgeable models are available. This rapid pace of social transmission suggests that learning can act to rapidly facilitate behavioural responses to environmental change [12].

We found that pay-off-biased learning and negative frequency dependence guided diffusion of panamá processing techniques in this group (table 2). These strategies are consistent with the observation that the rarest and most efficient panamá processing technique, canine seam, eventually became the most common. This was the case for most, but not all, naive and knowledgeable adults and subadults born after 2009 (figure 2). Juveniles born before 2009 did not use the canine seam technique (electronic supplementary material, figure S4; figure 2), probably because their mouths were not sufficiently large and strong.

Pay-off bias had the largest effect on the probability of choosing a behaviour, while negative frequency dependence may have prevented it from ever reaching fixation. Experimental evidence of wild animals using pay-off-biased learning has not been previously reported. Our finding of negative frequency-dependent learning suggests that capuchins bias their attention towards rare or novel behaviours—a type of neophilia.

While all adult individuals tried the canine seam technique, they typically settled on the technique or techniques that were most successful for them. Individuals who settled on the canine seam technique also sporadically tried other behaviours (electronic supplementary material, figure S4). This result is consistent with other research [60], suggesting that social learning guides exploration but personal experience strongly influences adoption.

While we found the strongest support for pay-off-biased learning, our modelling suggests that animals use multiple social learning strategies simultaneously, or that social biases and content biases might be equifinal. Age-biased learning also had support in the global model (table 2). This might be due to older individuals’ increased likelihood of being efficient panamá processors compared with juveniles, but the preferences for some individuals (JU and LN) to copy the techniques of the adults they commonly associate with who did not use canine seam (HE and MI, respectively) suggests otherwise.

Nevertheless, observational studies are always limited in their ability to distinguish some mechanisms from others. We believe that long-term field studies, field experiments and controlled captive experiments all have important and complementary roles to play.

### (b) Age predicts individual variation in social and individual learning

Individual variation in social learning may have meaningful evolutionary and social implications, yet remains poorly studied [13]. We found that younger individuals more heavily relied on social learning than older individuals (figure 4*b*) and that older individuals were less likely to observe conspecifics (electronic supplementary material, figure S5).

We also observed that older individuals were less likely to update information and had a greater attraction to previous experiences (figure 4*a*). This might be due to older individuals being less exploratory than younger individuals. One alternative explanation is that older individuals’ higher success rates at processing panamá provided them with higher-quality personal information to discern between the efficiency of varied processing techniques (electronic supplementary material, figure S4). This age structure in proclivity to learn socially suggests flexible learning strategies that change over development. Theory predicting and explaining such flexible variation waits to be constructed.

### (c) Statistical approach

Our analytical approach was designed around three important principles. First, it allows us to evaluate the possible influence of several different, theoretically plausible, social learning biases. Second, the framework combines social learning biases with a dynamic reinforcement model in which individuals remember and are influenced by past experience with different techniques. Third, the approach is multilevel, with each individual possessing its own parameters for relative use of each learning strategy. This allows us to evaluate heterogeneity and its contribution to population dynamics.

Our approach is distinct from looking for evidence of population-level learning dynamics consistent with the hypothesized learning strategy (i.e. sinosoidal curves and conformity) [24,61]. In our approach, any population-level patterns are consequences of inferred (and potentially different) strategies among individuals (visualized in electronic supplementary material, figure S3); they are not themselves used to make inferences about learning.

Our approach is most similar to network-based diffusion analysis (NBDA) [62,63]. In principle, our framework and NBDA can be analogized, despite differences in the details of modelled strategies, because both are multinomial time-series modelling frameworks that can be treated as both survival (time-to-event) or event history analyses. There are some notable differences in practice. Our approach differs from typically employed NBDA in that it (i) uses a full dynamic time series for available social information rather than a static social network, and (ii) emphasizes modelling the entire behavioural sequence including and beyond the first putative instance of social transmission. There is no reason in principle why ordinary NBDA models could not make similar use of these data, and recent advances [59] utilize dynamic social networks.

It is important to note that successfully fitting these dynamic, multilevel models benefits from recent advances in Monte Carlo algorithms. We used an implementation of Hamiltonian Monte Carlo (NUTS2) provided by Stan [34]. Our global model contains 231 parameters and would prove very challenging for older algorithms like Gibbs sampling. Hamiltonian Monte Carlo not only excels at high-dimension models, even with thousands of parameters, but it also provides greatly improved mixing diagnostics that allow us to have greater confidence in the correctness of the results, regardless of model complexity.

### (d) Implications for the origins and maintenance of traditions

This model suggests that pay-off-biased learning can cause the spread of a tradition. However, social learning may increase within-group homogeneity, while individual learning may act to decrease it [51]. Our findings are consistent with this idea. Limited transfer of individuals in xenophobic species like *Cebus* is exceptionally important in maintaining group-specific traditions for behaviours that differ in pay-off. However, this probably acts concordant with transmission biases. Variation might also be maintained due to biases for copying particular subsets of individuals (e.g. a particular age-class or kin group) in a stable social system. Migration of new individuals with more efficient behaviours could seed a new tradition in the group, the diffusion of which may be due to pay-off-biased learning.

### (e) Future directions

We have noted that equifinality might exist between learning strategies. On average, older individuals were better at opening panamá fruit. Perhaps individuals are biasing learning towards older individuals and acquiring the efficient techniques indirectly instead of turning attention towards the content of the behaviour. While we think this is probably not the case based on the evidence considered in this study, it is a possibility in all learning studies. In many cases, where we are interested in predicting the population dynamics of learning in a given context, the exact social learning strategy might not matter if it has the same dynamics and leads to the same frequency in a population. Many learning strategies are likely to be equifinal under the right social conditions. However, the exact nature of the cognitive mechanisms of the learning strategies organisms employ, and the social factors which indirectly structure learning become important when we wish to use social learning in applied contexts. Further theoretical and empirical explorations of social learning need to address that learning is a two-stage process: one of assortment and one of information use.

An important aspect of learning that we have neglected is the endogeneity of social information. Our statistical models evaluated how individuals use information they observed. However, before individuals acquire social information, they make the decision to observe others. Future analyses will evaluate who individuals choose to bias attention towards when in the proximity of potential demonstrators to see how positive assortment due to social preferences, rank or food sharing might structure opportunities for social learning and affect the establishment and maintenance of traditions.

Most models of social learning in the evolutionary anthropology and animal behaviour literature assume a randomly assorted population. However, non-random assortment occurs before information is acquired in a population, and it can drastically affect social learning and cultural dynamics. Sometimes this assortment may be an adaptive heuristic, such as deciding to bias attention. Other times, it may be an indirect consequence of social behaviour, such as avoidance of a potentially dangerous demonstrator [15]. Asymmetrical age structure in a population may also make the behavioural variants in the population non-random when learning abilities are constrained by skill and developing cognition [64]. Social networks can also change drastically over development, opening up avenues for new possible learning strategies. Some learning strategies might be difficult to tease apart in small, non-diverse social systems. If juveniles engage in kin-biased learning [65], but only interact with their kin group, how are we to discern kin-biased learning from linear imitation or conformity, and under what conditions does this distinction matter?

## Supplementary Material

Supplemental Materials

## Supplementary Material

Figure S1

## Supplementary Material

Figure S2

## Supplementary Material

Figure S3

## Supplementary Material

Figure S4

## Supplementary Material

Figure S5

## Data accessibility

Data and code for models, simulations and graphs are available at https://github.com/bjbarrett/panama1.

## References

[1] Fragaszy DM, Perry S 2003 The biology of traditions: models and evidence. Cambridge, UK: Cambridge University Press.

[2] Kawamura S 1959 The process of sub-culture propagation among Japanese macaques. Primates 2, 43–60. (10.1007/BF01666110)

[3] Marler P, Tamura M 1964 Culturally transmitted patterns of vocal behavior in sparrows. Science 146, 1483–1486. (10.1126/science.146.3650.1483) 14208581

[4] Bonner JT 1983 The evolution of culture in animals, Reprint edition Princeton, NJ: Princeton University Press.

[5] Whiten A, Goodall J, McGrew WC, Nishida T, Reynolds V, Sugiyama Y, Tutin CEG, Wrangham RW, Boesch C 1999 Cultures in chimpanzees. Nature 399, 682–685. (10.1038/21415) 10385119

[6] van Schaik CP, Ancrenaz M, Borgen G, Galdikas B, Knott CD, Singleton I, Suzuki A, Utami SS, Merrill M 2003 Orangutan cultures and the evolution of material culture. Science 299, 102–105. (10.1126/science.1078004) 12511649

[7] Perry S 2003 Social conventions in wild white-faced capuchin monkeys: evidence for traditions in a neotropical primate. Curr. Anthropol. 44, 241–268. (10.1086/345825)

[8] Whitehead H 1998 Cultural selection and genetic diversity in matrilineal whales. Science 282, 1708–1711. (10.1126/science.282.5394.1708) 9831562

[9] Rendell LE, Whitehead H 2001 Culture in whales and dolphins. Behav. Brain Sci. 24, 309–382. (10.1017/S0140525X0100396X) 11530544

[10] Boyd R, Richerson PJ 1985 Culture and the evolutionary process. Chicago, IL: University of Chicago Press.

[11] Laland KN 2004 Social learning strategies. Anim. Learn. Behav. 32, 4–14. (10.3758/BF03196002) 15161136

[12] Rendell L 2010 Why copy others? Insights from the social learning strategies tournament. Science 328, 208–213. (10.1126/science.1184719) 20378813 PMC2989663

[13] Mesoudi A, Chang L, Dall SRX, Thornton A 2016 The evolution of individual and cultural variation in social learning. Trends Ecol. Evol. (Amst.) 31, 215–225. (10.1016/j.tree.2015.12.012) 26775795

[14] Farine DR, Spencer KA, Boogert NJ 2015 Early-life stress triggers juvenile zebra finches to switch social learning strategies. Curr. Biol. 25, 2184–2188. (10.1016/j.cub.2015.06.071) 26212879 PMC4540255

[15] Russon AE 2002 Comparative developmental perspectives on culture: the great apes. In Between culture and biology: perspectives on ontogenetic development (eds Keller H, Poortinga YH, Schlmerich A), pp. 30–56. Cambridge, UK: Cambridge University Press.

[16] McElreath R, Lubell M, Richerson PJ, Waring TM, Baum W, Edsten E, Efferson C, Paciotti B 2005 Applying evolutionary models to the laboratory study of social learning. Evol. Hum. Behav. 26, 483–508. (10.1016/j.evolhumbehav.2005.04.003)

[17] McElreath R, Bell AV, Efferson C, Lubell M, Richerson PJ, Waring T 2008 Beyond existence and aiming outside the laboratory: estimating frequency-dependent and pay-off-biased social learning strategies. Phil. Trans. R. Soc. B 363, 3515–3528. (10.1098/rstb.2008.0131) 18799416 PMC2607339

[18] Henrich J, Boyd R 1998 The evolution of conformist transmission and the emergence of between-group differences. Evol. Hum. Behav. 19, 215–241. (10.1016/S1090-5138(98)00018-X)

[19] Nakahashi W, Wakano JY, Henrich J 2012 Adaptive social learning strategies in temporally and spatially varying environments. Hum. Nat. 23, 386–418. (10.1007/s12110-012-9151-y) 22926986

[20] Day RL, MacDonald T, Brown C, Laland KN, Reader SM 2001 Interactions between shoal size and conformity in guppy social foraging. Anim. Behav. 62, 917–925. (10.1006/anbe.2001.1820)

[21] Whitehead H, Richerson PJ 2009 The evolution of conformist social learning can cause population collapse in realistically variable environments. Evol. Hum. Behav. 30, 261–273. (10.1016/j.evolhumbehav.2009.02.003)

[22] Baldini R 2013 Two success-biased social learning strategies. Theor. Popul. Biol. 86, 43–49. (10.1016/j.tpb.2013.03.005) 23587700

[23] van de Waal E, Borgeaud C, Whiten A 2013 Potent social learning and conformity shape a wild primate's foraging decisions. Science 340, 483–485. (10.1126/science.1232769) 23620053

[24] Aplin LM, Farine DR, Morand-Ferron J, Cockburn A, Thornton A, Sheldon BC 2015 Experimentally induced innovations lead to persistent culture via conformity in wild birds. Nature 518, 538–541. (10.1038/nature13998) 25470065 PMC4344839

[25] Oppenheimer JR 1968 Behavior and ecology of the white-faced monkey: Cebus capucinus, on Barro Colorado Island, C.Z. Champaign, IL: University of Illinois.

[26] Fragaszy DM, Visalberghi E, Fedigan LM 2004 The complete capuchin: the biology of the genus Cebus. Cambridge, UK: Cambridge University Press.

[27] Jerison H 1973 Evolution of the brain and intelligence. San Diego, CA: Academic Press.

[28] Rose LM, Perry S, Panger MA, Jack K, Manson JH, Gros-Louis J, Mackinnon KC, Vogel E 2003 Interspecific interactions between *Cebus capucinus* and other species: data from three Costa Rican sites. Int. J. Primatol. 24, 759–796.

[29] Panger MA, Perry S, Rose LM, Gros-Louis J, Vogel E, MacKinonn KC, Baker M 2002 Cross-site differences in foraging behavior of white-faced capuchins (*Cebus capucinus*). Am. J. Phys. Anthropol. 119, 52–66. (10.1002/ajpa.10103) 12209573

[30] Perry S 2009 Conformism in the food processing techniques of white-faced capuchin monkeys (*Cebus capucinus*). Anim. Cogn. 12, 705–716. (10.1007/s10071-009-0230-3) 19455357 PMC2728904

[31] O'Malley RC, Fedigan LM 2005 Evaluating social influences on food-processing behavior in white-faced capuchins (*Cebus capucinus*). Am. J. Phys. Anthropol. 127, 481–491. (10.1002/ajpa.20095) 15693024

[32] Perry S 2011 Social traditions and social learning in capuchin monkeys (*Cebus*). Phil. Trans. R. Soc. B 366, 988–996. (10.1098/rstb.2010.0317) 21357221 PMC3049088

[33] Sol D, Sayol F, Ducatez S, Lefebvre L 2016 The life-history basis of behavioural innovations. Phil. Trans. R. Soc. B 371, 20150187 (10.1098/rstb.2015.0187) 26926277 PMC4780529

[34] Stan Development Team. 2016 RStan: the R interface to Stan., v. 2.14.0. See http://mc-stan.org/rstan.html.

[35] Perry S, Godoy I, Lammers W 2012 The Lomas Barbudal Monkey Project: two decades of research on *Cebus capucinus*. In Long-term field studies of primates (eds Kappeler PM, Watts DP), pp. 141–163. Berlin, Germany: Springer.

[36] Frankie GW, Vinson SB, Newstrom LE, Barthell JF 1988 Nest site and habitat preferences of *Centris* bees in the Costa Rican dry forest. Biotropica 20, 301–310. (10.2307/2388320)

[37] Perry S, Ordoñez Jiménez JC 2006 The effects of food size, rarity, and processing complexity on white-faced capuchins visual attention to foraging conspecifics. In Feeding ecology in apes and other primates (eds Hohmann G, Robbins MM, Boesch C), vol. 48, pp. 203–234. Cambridge, UK: Cambridge University Press.

[38] Janzen DH 1972 Escape in space by *Sterculia apetala* seeds from the bug *Dysdercus fasciatus* in a Costa Rican deciduous forest. Ecology 53, 350–361. (10.2307/1934092)

[39] Camerer C, Hua Ho T 1999 Experience-weighted attraction learning in normal form games. Econometrica 67, 827–874. (10.1111/1468-0262.00054)

[40] Hoppitt W, Laland KN 2013 Social learning: an introduction to mechanisms, methods, and models. Princeton, NJ: Princeton University Press.

[41] Kendal R, Hopper LM, Whiten A, Brosnan SF, Lambeth SP, Schapiro SJ, Hoppitt W 2015 Chimpanzees copy dominant and knowledgeable individuals: implications for cultural diversity. Evol. Hum. Behav. 36, 65–72. (10.1016/j.evolhumbehav.2014.09.002) 27053916 PMC4820294

[42] Baldini R 2012 Success-biased social learning: cultural and evolutionary dynamics. Theor. Popul. Biol. 82, 222–228. (10.1016/j.tpb.2012.06.005) 22743216

[43] Pike TW, Kendal JR, Rendell LE, Laland KN 2010 Learning by proportional observation in a species of fish. Behav. Ecol. 21, 570–575. (10.1093/beheco/arq025)

[44] Ottoni EB, Resende BDd, Izar P 2005 Watching the best nutcrackers: what capuchin monkeys (*Cebus apella*) know about others’ tool-using skills. Anim. Cogn. 8, 215–219. (10.1007/s10071-004-0245-8) 15719240

[45] Henrich J, Gil-White FJ 2001 The evolution of prestige: freely conferred deference as a mechanism for enhancing the benefits of cultural transmission. Evol. Hum. Behav. 22, 165–196. (10.1016/S1090-5138(00)00071-4) 11384884

[46] Horner V, Proctor D, Bonnie KE, Whiten A, Waal FBMd 2010 Prestige affects cultural learning in chimpanzees. PLoS ONE 5, e10625 (10.1371/journal.pone.0010625) 20502702 PMC2873264

[47] van de Waal E, Renevey N, Favre CM, Bshary R 2010 Selective attention to philopatric models causes directed social learning in wild vervet monkeys. Proc. R. Soc. B 277, 2105–2111. (10.1098/rspb.2009.2260) PMC2880145 20236972

[48] McElreath R, Strimling P 2008 When natural selection favors imitation of parents. Curr. Anthropol. 49, 307–316. (10.1086/524364)

[49] Mazur R, Seher V 2008 Socially learned foraging behaviour in wild black bears, *Ursus americanus*. Anim. Behav. 75, 1503–1508. (10.1016/j.anbehav.2007.10.027)

[50] Müller CA, Cant MA 2010 Imitation and traditions in wild banded mongooses. Curr. Biol. 20, 1171–1175. (10.1016/j.cub.2010.04.037) 20605459

[51] Thornton A, Clutton-Brock T 2011 Social learning and the development of individual and group behaviour in mammal societies. Phil. Trans. R. Soc. B 366, 978–987. (10.1098/rstb.2010.0312) 21357220 PMC3049086

[52] Chou LS, Richerson PJ 1992 Multiple models in social transmission of food selection by Norway rats, *Rattus norvegicus*. Anim. Behav. 44, 337–343. (10.1016/0003-3472(92)90039-C)

[53] Whiten A, Horner V, de Waal FBM 2005 Conformity to cultural norms of tool use in chimpanzees. Nature 437, 737–740. (10.1038/nature04047) 16113685

[54] Dindo M, Whiten A, de Waal FBM 2009 In-group conformity sustains different foraging traditions in capuchin monkeys (*Cebus apella*). PLoS ONE 4, e7858 (10.1371/journal.pone.0007858) 19924242 PMC2773420

[55] Pike TW, Laland KN 2010 Conformist learning in nine-spined sticklebacks’ foraging decisions. Biol. Lett. 6, 466–468. (10.1098/rsbl.2009.1014) 20129948 PMC2936200

[56] R Core Team. 2016 R: a language and environment for statistical computing. Vienna, Austria: R Foundation for Statistical Computing.

[57] Watanabe S 2010 Asymptotic equivalence of Bayes cross validation and widely applicable information criterion in singular learning theory. J. Mach. Learn. Res. 11, 3571–3594.

[58] Fragaszy D, Visalberghi E 2004 Socially biased learning in monkeys. Anim. Learn. Behav. 32, 24–35. (10.3758/BF03196004) 15161138

[59] Hobaiter C, Poisot T, Zuberbühler K, Hoppitt W, Gruber T 2014 Social network analysis shows direct evidence for social transmission of tool use in wild chimpanzees. PLoS Biol. 12, e1001960 (10.1371/journal.pbio.1001960) 25268798 PMC4181963

[60] Galef BG, Whiskin EE 2001 Interaction of social and individual learning in food preferences of Norway rats. Anim. Behav. 62, 41–46. (10.1006/anbe.2000.1721)

[61] Henrich J 2001 Cultural transmission and the diffusion of innovations: adoption dynamics indicate that biased cultural transmission is the predominate force in behavioral change. Am. Anthropol. 103, 992–1013. (10.1525/aa.2001.103.4.992)

[62] Hoppitt W, Laland KN 2011 Detecting social learning using networks: a users guide. Am. J. Primatol. 73, 834–844. (10.1002/ajp.20920) 21246592

[63] Allen J, Weinrich M, Hoppitt W, Rendell L 2013 Network-based diffusion analysis reveals cultural transmission of lobtail feeding in humpback whales. Science 340, 485–488. (10.1126/science.1231976) 23620054

[64] Russon AE 2003 Developmental perspectives on great ape traditions. In The biology of traditions: models and evidence (eds Fragaszy DM, Perry S), pp. 329–364. Cambridge, UK: Cambridge University Press.

[65] Kline MA, Boyd R, Henrich J 2013 Teaching and the life history of cultural transmission in Fijian villages. Hum. Nat. 24, 351–374. (10.1007/s12110-013-9180-1) 24096923

