# Supplementary material for: Pay-off-biased social learning underlies the diffusion of novel extractive foraging traditions in a wild primate
Source: Proc Biol Sci. 2017 Jun 7;284(1856):20170358. doi: 10.1098/rspb.2017.0358 (PMC5474070; doi:10.1098/rspb.2017.0358)
Supplement: Supplemental Materials [file rspb20170358supp1.pdf]

SUPPLEMENTAL MATERIALS FOR *PAYOFF-BIASED SOCIAL LEARNING UNDERLIES THE DIFFUSION OF NOVEL EXTRACTIVE FORAGING TRADITIONS IN A WILD PRIMATE*

BRENDAN J BARRETT, RICHARD L MCELREATH, AND SUSAN E PERRY

Supplemental Materials

Detail about the fieldsite: Lomas Barbudal. RBLB has been the location of the Lomas Barbudal monkey project which, between May 1990 and the end of Feb 2015, collected 96,250 hours of observational behavioural data on 538 monkeys, and determined genetic parentage for most of these individuals. The habitat is neotropical dry forest, including much riparian forest, and is marked by a rainy wet season from May-November with 1,000-2,000 mm of rain and a dry season marked by little to no rain and the deciduous shedding of the leaves of most tree species to cope with water limitation [36]. Long-term research at this field site has made it possible to document many unique aspects of capuchin biology and behaviour that would not have been possible with short term observations (i.e. < 5-10 years), including many social conventions, behavioural traditions, rare dispersal events, and the study covered in this manuscript [37].

Fruit Collection. The locations of 79 mature panamá trees near RBLB were recorded using GPS and compiled from phenological transects, the recollection of long term researchers, and surveys in areas conducive to their ecology. Panamá trees are easy to locate in the forest as they are often large (up to 35 m tall), primarily grow near limited water sources, and have conspicuous buttresses and large palmate leaves that densely litter the forest floor. Panamá fruits used in the study were collected at trees where fruits showed evidence of capuchin foraging. Fruits chosen were of early stage ripeness, had not dehisced, had mature-sized seeds, showed no evidence of physical tampering by animals, and were used within 48 hours of collection.

Additional Behavioural Data Collection. Observers were trained for at least one month on monkey identification in the field using facial recognition, size, and unique marks, and also memorized an ethogram of panamá foraging and social behaviour. We recorded the panamá processing actions of the focal individual, and the focal's interactions with potential audience members. Monkeys were assumed to be observing processing if they stopped to focused their gaze on the processing individual for more than 3 seconds. If they briefly glanced at the foraging individual, or focused their stare on them while the demonstrator was ingesting seeds but did not observe processing, they were assumed to be not acquiring information about food processing.The location of potential audience members relative to the focal individual was recorded as one of: in contact, <1 , <5, <10, or <20 adult monkey lengths. One adult capuchin monkey length is approximately 0.75 m. Additional social behaviours were recorded, including submissive and aggressive interactions, tolerated food handling, untolerated food theft, and scrounging. Exceptionally low-ranking individuals tended not to grab fruits from the processing platform when in proximity to high-rankers who were not in possession of a fruit. Once a fruit was in the hands of a monkey, it was rarely aggressively usurped (<10 times) by other monkeys regardless of age or rank asymmetries. Scrounging typically occurred only if the foraging individuals dropped any seeds or decided not to eat all the seeds in the fruit. After each experimental bout, fruits were recovered and photographed to note

technique-specific markings and validate the observations of researchers. Only 4 panamá processing events were not viewed by the observer due to an animal darting away quickly with a fruit. Unfortunately, an estimated 114 processing bouts are missing from this data set collected on days 39,40,42,43,45,48, and 49 that were lost by a research assistant. While we have photographs of most of these processed fruits, we did not analyze them or impute processing times.

Table S1. Counts and percentages of total foraging time spent consuming panamá for RBLB study groups collected between July 2003 and December 2011. Each count is a activity point sample collected every 2.5 minutes from a 10 minute continuous focal follow. Rafiki's group was not observed foraging for panamá during focal follows since their late 2007 fission from Pelon group, however there are 14 panamá trees within their territory in which they have been observed foraging.

**Group**	**# times foraging**	**# times foraging panamá**	**% foraging time spent on panamá**
Abby's	34,263	78	0.23
Cupie's	4,431	3	0.07
Pelon	36,054	10	0.03
Flakes	17,200	0	0.00
Lost Boys	4,357	50	1.15
Musketeers	8,866	17	0.19
Newman's	3,951	48	1.21
Rafiki's	6,745	0	0.00
Rambo's	32,893	144	0.44
Splinter	2,944	18	0.61

Table S2. Summary statistics for the 7 panamá processing techniques observed in this study. Mean and median duration presented in seconds.

**Technique**	**Description**	**Mean**	**Median**	**% Open**	**N**
Back attack	peel fibres off back from fruit with seam facing away from mouth, bite to pop open at seam	169.0	119.0	51.1	176
Bite and pop	bite opposite corners of each fruit forcefully, bite to pop open at seam	49.7	29	37.8	283
Canine seam	hold fruit perpendicular to mouth, insert upper and lower canines into seam to split open	70.5	42	88.5	511
Chew hole	chew hole or rip fibres off fruit at corner, back, or side, seam not chewed	330.5	211.5	65.5	247
Pound	pound fruit on hard substrate	n/a	n/a	0	15
Scrub	scrub fruit on hard substrate	n/a	n/a	0	5
Seam Strip	hold fruit parallel to mouth, strip fibres off along the seam, bite to pop open at seam	130.6	211.5	65.0	200
All techs		131.5	95	65.6	1437

Table S3. Widely applicable information criteria (WAIC) estimates, WAIC model weights (wWAIC), posterior median (PME), posterior standard deviation (SD), and standard deviation of varying effects for individuals (σ) for all evaluated models.

**Model**	Global		Freq. Dep.		Payoff-bias		Age Bias		Cohort Bias		Rank Bias		Kin Bias		Individual		Null
**WAIC**	4005.12		4010.77		4021.88		4031.79		4053.04		4054.69		4060.97		4071.97		5526.40
**wWAIC**	0.94		0.06		<0.01		<0.01		<0.01		<0.01		<0.01		<0.01		<0.01
	PME	SD	PME	SD	PME	SD	PME	SD	PME	SD	PME	SD	PME	SD	PME	SD	
λ	20.97	*1.11*	21.03	*1.08*	23.22	*1.41*	22.58	*1.36*	22.42	*1.27*	22.44	*1.31*	22.33	*1.32*	19.51	*0.96*	
ϕ	0.15	*0.03*	0.16	*0.03*	0.15	*0.03*	0.15	*0.03*	0.15	*0.03*	0.15	*0.03*	0.15	*0.03*	0.13	*0.03*	
γ	0.14	*0.03*	0.14	*0.03*	0.15	*0.05*	0.10	*0.04*	0.09	*0.04*	0.09	*0.03*	0.08	*0.03*			
f	0.38	*0.28*	0.53	*0.12*													
β_{pay}	1.02	*0.84*			3.49	*0.89*											
β_{kin}	0.19	*0.93*											0.35	*0.99*			
β_{rank}	-0.11	*0.91*									1.12	*0.96*					
β_{coho}	0.48	*0.93*							1.07	*1.03*							
β_{age}	0.69	*0.92*					2.11	0.83									
μ_{age} (ϕ)	-0.11	*0.03*	-0.11	*0.03*	-0.09	*0.03*	-0.10	*0.03*	-0.09	*0.03*	-0.09	*0.03*	-0.09	*0.03*	-0.08	*0.03*	
μ_{age} (γ)	-0.10	*0.05*	-0.09	*0.05*	-0.01	*0.05*	0.02	*0.06*	0.02	*0.06*	0.03	*0.05*	0.03	*0.05*			
σ_ϕ	0.66	*0.03*	0.67	*0.03*	0.66	*0.03*	0.66	*0.03*	0.67	*0.03*	0.67	*0.03*	0.67	*0.03*	0.67	*0.04*	
σ_γ	0.69	*0.06*	0.68	*0.06*	0.72	*0.07*	0.74	*0.07*	0.69	*0.09*	0.67	*0.09*	0.62	*0.08*			
σ_f	1.29	*1.19*	1.37	*1.18*													
$\sigma_{\beta_{pay}}$	0.28	*0.42*			0.71	*0.84*											
$\sigma_{\beta_{kin}}$	0.25	*0.40*											0.22	*0.35*			
$\sigma_{\beta_{rank}}$	0.26	*0.42*									0.29	*0.51*					
$\sigma_{\beta_{coho}}$	0.26	*0.40*							0.52	*0.83*							
$\sigma_{\beta_{age}}$	0.25	*0.38*					0.84	*0.70*									

TABLE S4. Posterior median estimates (PME) and standard deviations (SD) for global models evaluating social information from temporal windows of the previous 7, 21, and 28 days. Predictions are comparable to one another and to those presented in the main paper with a width of the previous 14 days.

Social Info Window Width	7 days		21 days		28 days	
Parameter	PME	SD	PME	SD	PME	SD
λ	20.98	*1.10*	20.81	*1.10*	20.95	*1.13*
ϕ	0.16	*0.03*	0.15	*0.03*	0.15	*0.03*
γ	0.14	*0.03*	0.16	*0.04*	0.14	*0.04*
f	0.39	*0.28*	0.35	*0.21*	0.39	*0.28*
β_{pay}	1.03	*0.86*	1.16	*0.73*	1.04	*0.87*
β_{kin}	0.17	*0.96*	0.05	*0.93*	0.19	*0.95*
β_{rank}	-0.10	*0.93*	0.08	*0.90*	-0.10	*0.92*
β_{coho}	0.47	*0.95*	0.32	*0.91*	0.48	*0.93*
β_{age}	0.68	*0.88*	0.87	*0.87*	0.67	*0.90*
μ_{age} (ϕ)	-0.11	*0.03*	-0.11	*0.03*	-0.11	*0.03*
μ_{age} (γ)	-0.10	*0.05*	-0.12	*0.05*	-0.10	*0.05*
σ_ϕ	0.67	*0.03*	0.67	*0.03*	0.66	*0.03*
σ_γ	0.69	*0.06*	0.70	*0.06*	0.69	*0.06*
σ_f	1.28	*1.17*	1.29	*0.77*	1.28	*0.96*
$\sigma_{\beta_{pay}}$	0.28	*0.43*	0.38	*0.54*	0.28	*0.42*
$\sigma_{\beta_{kin}}$	0.24	*0.39*	0.26	*0.45*	0.25	*0.37*
$\sigma_{\beta_{rank}}$	0.26	*0.42*	0.26	*0.41*	0.26	*0.43*
$\sigma_{\beta_{coho}}$	0.25	*0.40*	0.25	*0.40*	0.26	*0.38*
$\sigma_{\beta_{age}}$	0.27	*0.38*	0.24	*0.36*	0.25	*0.38*

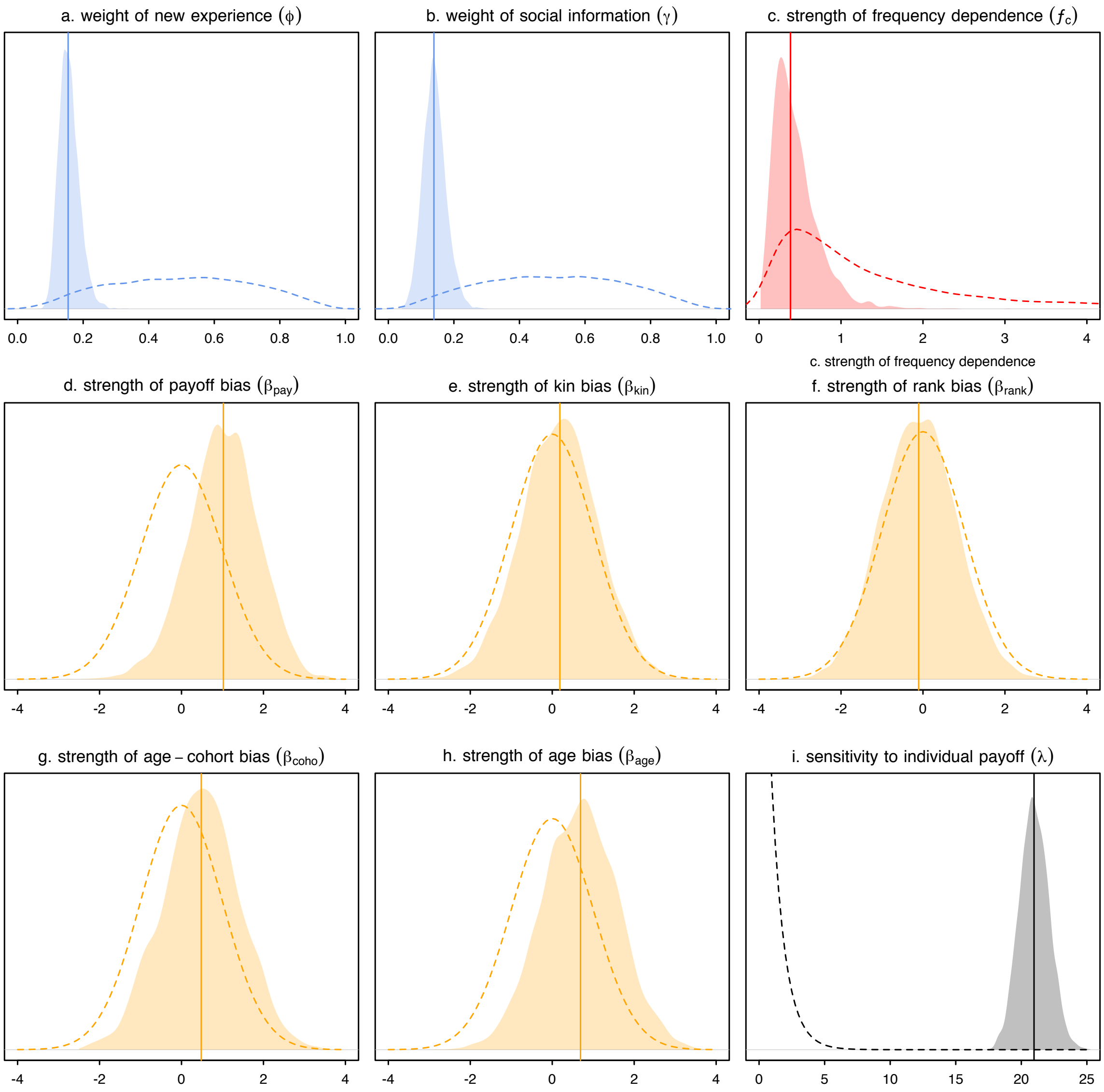

Figure S1. Posterior distributions of the main effects of the estimated parameters for the global model. Vertical solid lines lie at posterior mean. Dashed curves are the prior probabilities used to estimate each parameter.

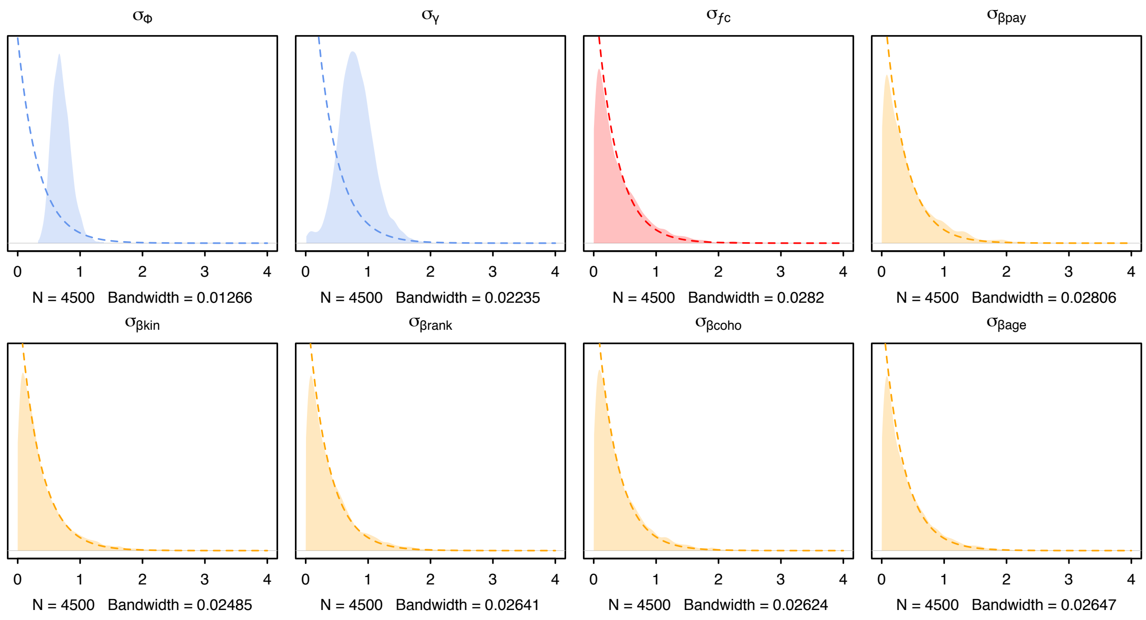

FIGURE S2. Posterior distributions of estimates of σ, the standard deviation of varying effects across all individuals. Dashed curves are the prior probabilities used to estimate each parameter.

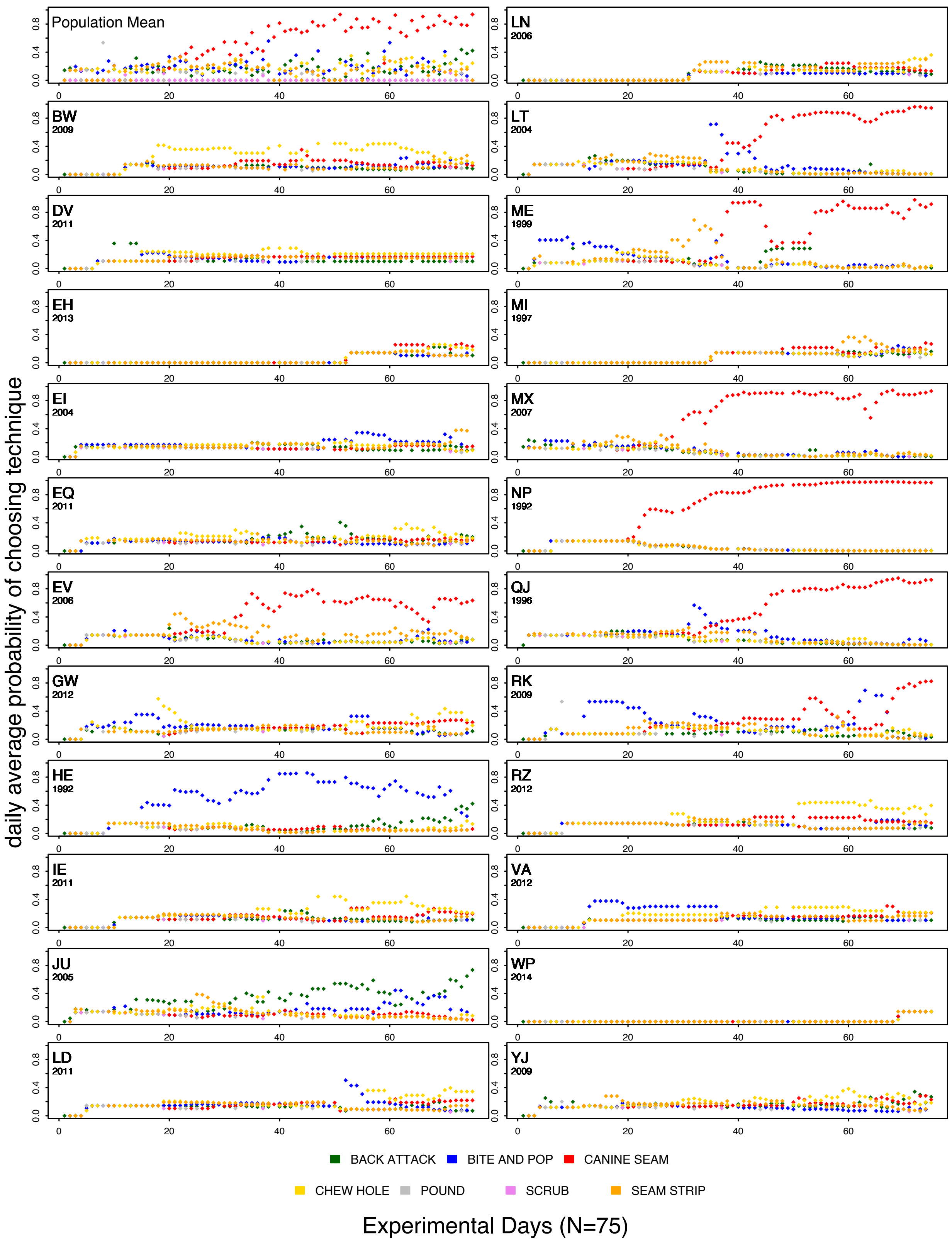

FIGURE S3. Daily average mean probability of choosing a processing technique. All plots are for each unique individual with the exception of the top left plot which is the population mean average. ID codes are in upper left hand corner. Numbers below ID codes are year of birth for each individual. The top left is population mean panel is a model estimate of the commonly observed diffusion curve seen in many social learning studies; note how it does not accurately represent most individuals in the population.

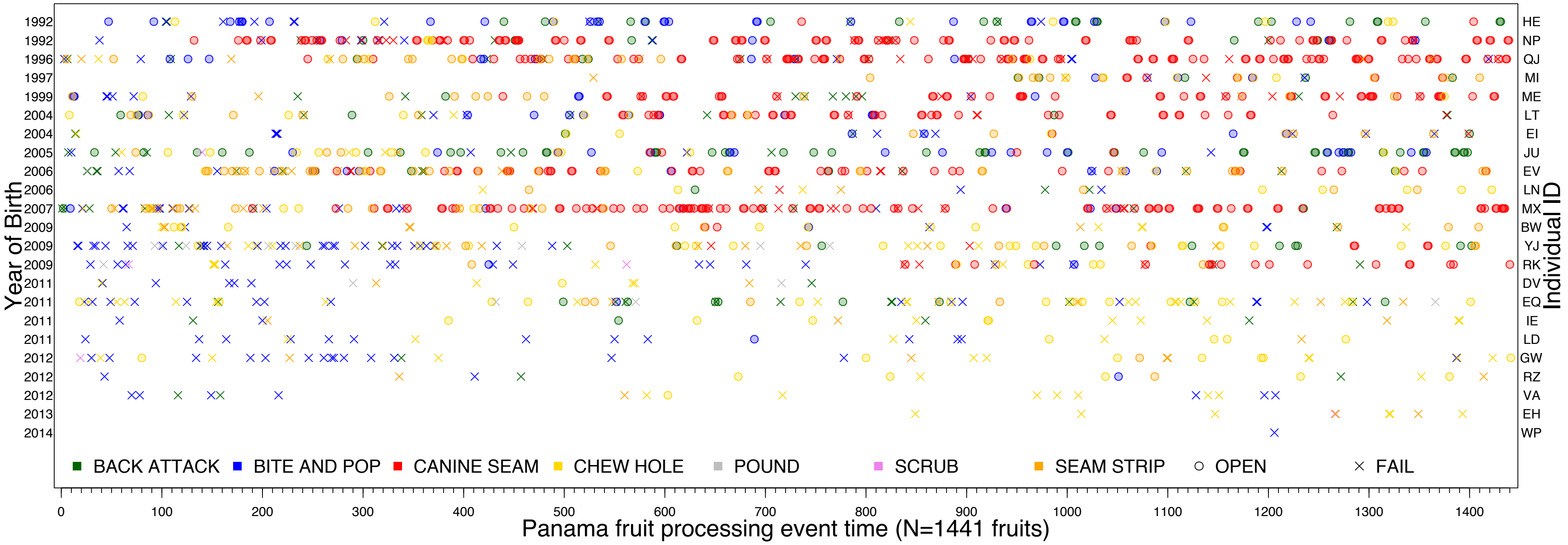

FIGURE S4. Techniques observed during experiment. Rows are unique individuals, from older (top) to younger (bottom). X-axis is event time. Each color represents a processing technique. ○ indicate successes. × indicate failures. The most successful technique in red (canine seam) diffused to older members of the population. Younger individuals did not use canine seam, had more failed attempts, and fewer processing attempts. Points are raw data.

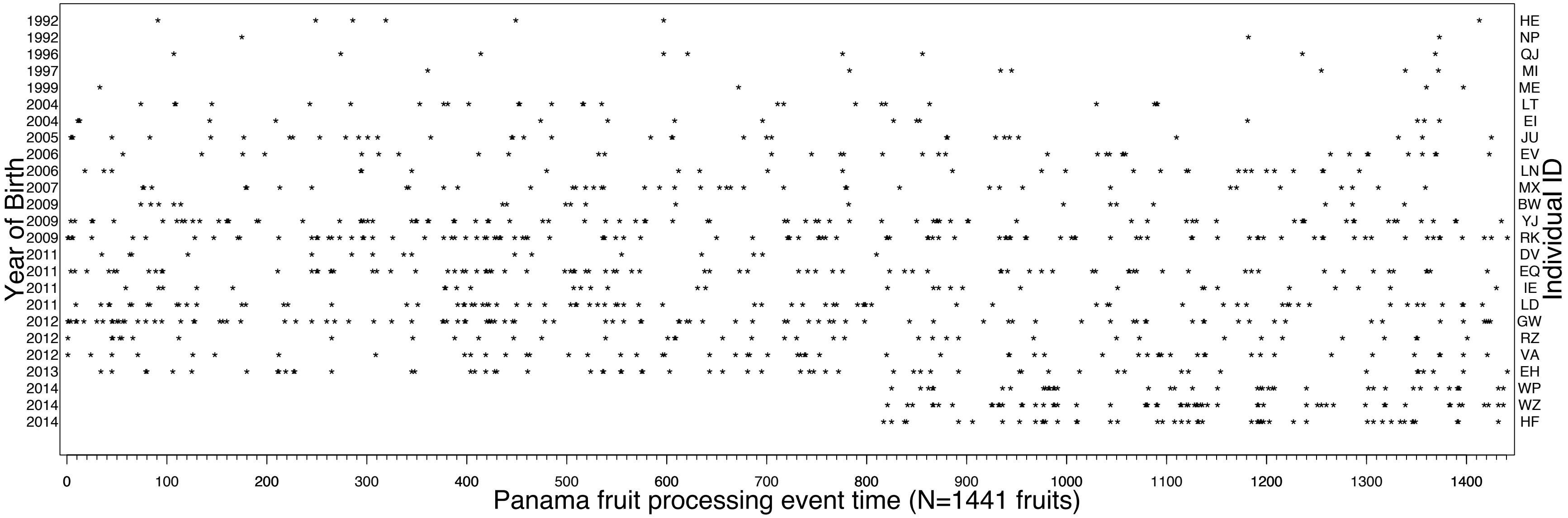

Figure S5. Plot of incidences of focused, sustained observation of another capuchin (in each row) observing a panamá foraging demonstrator. Older individuals are at the top of the graph. The x-axis is the event time of each fruit processed by a single individual. Each star indicated an observation event by the individual labelled on the y-axis.

Additional Acknowledgements: For long term data collected at Lomas, we are grateful to H. Gilkenson, W. Lammers, C. Dillis, M. Corrales, and R. Popa, for helping manage the field site. We thank the following field assistants assisted with data collection, each contributing a year or more of data to the Lomas Barbudal Monkey Project data set: L. Beaudrot, M. Bergstrom, R. Berl, A. Bjorkman, L. Blankenship, T. Borcuch, J. Broesch, J. Butler, F. Campos, C. Carlson, S. Caro, M. Corrales, N. Donati, C. Dillis, R. Dower, K. Feilen, K. Fisher, A. Fuentes J., M. Fuentes A., C. Gault, H. Gilkenson, I. Gottlieb, L. Hack, S. Herbert, C. Hirsch, C. Holman, S. Hyde, L. Johnson, W. Lammers, S. Lee, S. Leinwand, K. Kajokaite, M. Kay, E. Kennedy, D. Kerhoas-Essens, E. Johnson, S. Kessler, S. MacCarter, J. Manson, W. Meno, C. Mitchell, Y. Namba, A. Neyer, C. O'Connell, J.C.Ordoñez J., R. Popa, K. Ratliff , H. Ruffler, S. Sanford, M. Saul, I. Schamberg, C. Schmitt, A. Scott, J. Verge, A. Walker-Bolton, and E. Williams. BB and RM thank E. and A. van Halen for moral support while drafting this manuscript.
