# Supplementary material for: Pay-off-biased social learning underlies the diffusion of novel extractive foraging traditions in a wild primate
Source: Proc Biol Sci. 2017 Jun 7;284(1856):20170358. doi: 10.1098/rspb.2017.0358 (PMC5474070; doi:10.1098/rspb.2017.0358)
Supplement: Figure S1 [file rspb20170358supp2.pdf]

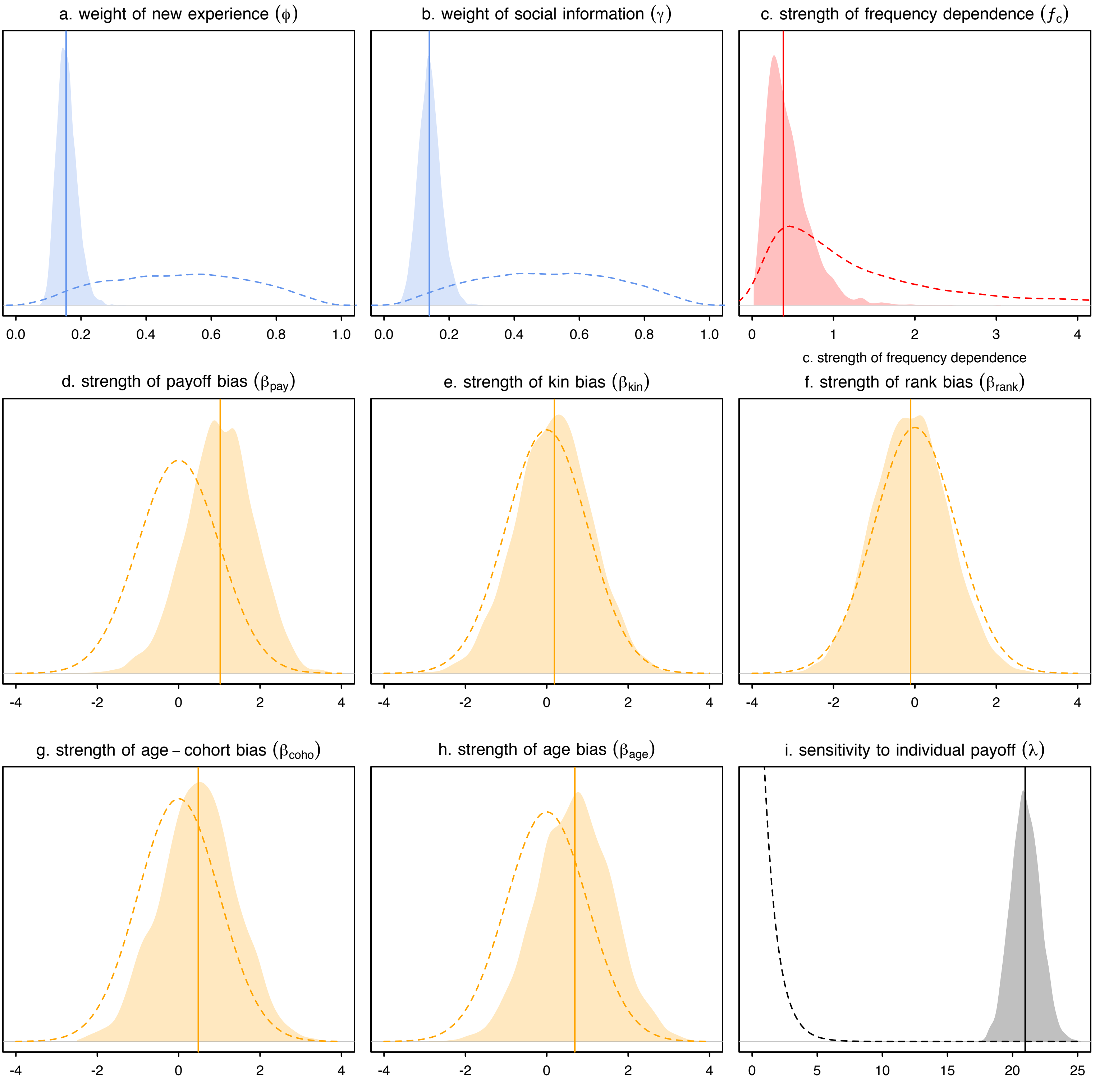

a. weight of new experience ($\phi$)
0.0 0.2 0.4 0.6 0.8 1.0
b. weight of social information ($\gamma$)
0.0 0.2 0.4 0.6 0.8 1.0
c. strength of frequency dependence ($f_c$)
0 1 2 3 4
c. strength of frequency dependence
d. strength of payoff bias ($\beta_{pay}$)
-4 -2 0 2 4
e. strength of kin bias ($\beta_{kin}$)
-4 -2 0 2 4
f. strength of rank bias ($\beta_{rank}$)
-4 -2 0 2 4
g. strength of age – cohort bias ($\beta_{coho}$)
-4 -2 0 2 4
h. strength of age bias ($\beta_{age}$)
-4 -2 0 2 4
i. sensitivity to individual payoff ($\lambda$)
0 5 10 15 20 25
