# Supplementary material for: Pay-off-biased social learning underlies the diffusion of novel extractive foraging traditions in a wild primate
Source: Proc Biol Sci. 2017 Jun 7;284(1856):20170358. doi: 10.1098/rspb.2017.0358 (PMC5474070; doi:10.1098/rspb.2017.0358)
Supplement: Figure S2 [file rspb20170358supp3.pdf]

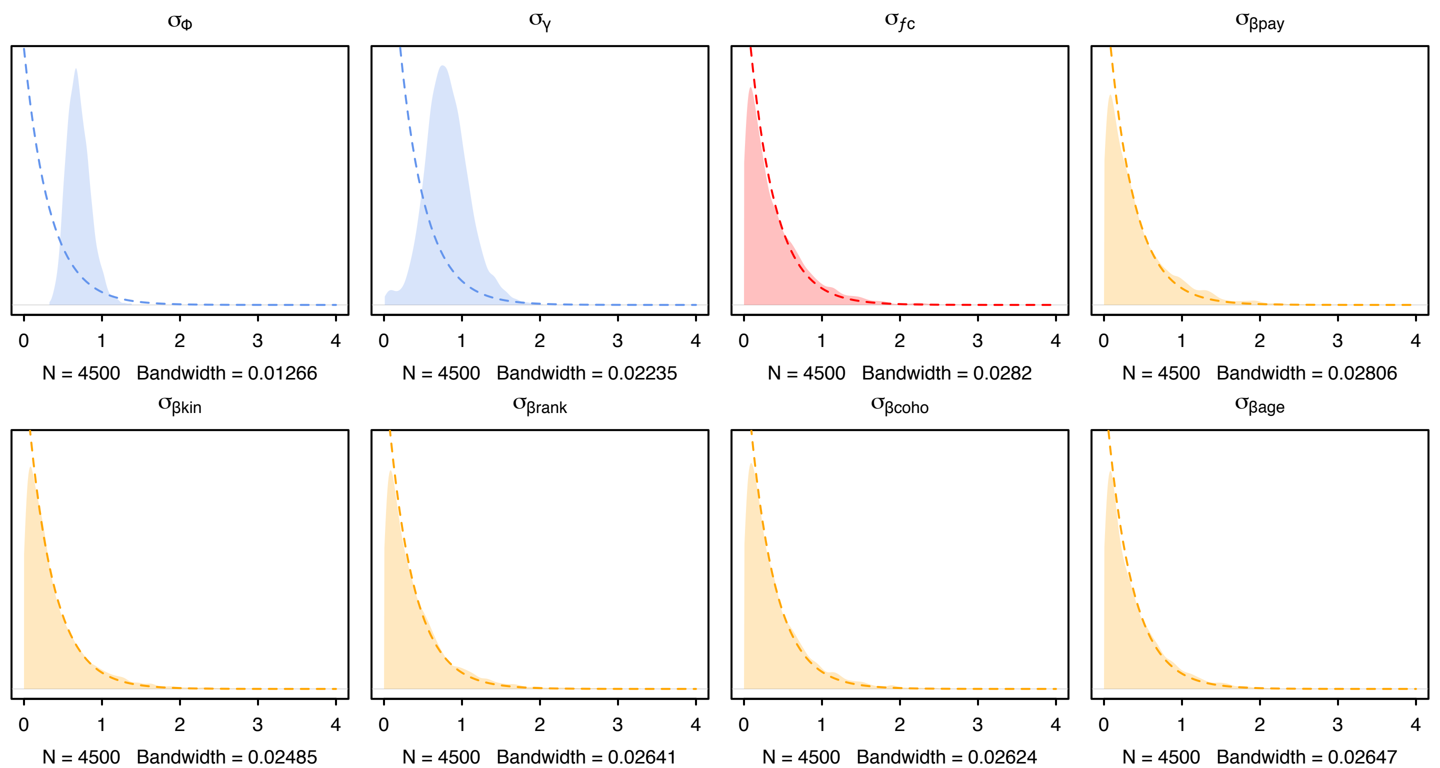
σ_{Φ}
N = 4500 Bandwidth = 0.01266
σ_{γ}
N = 4500 Bandwidth = 0.02235
σ_{fc}
N = 4500 Bandwidth = 0.0282
$\sigma_{\beta pay}$
N = 4500 Bandwidth = 0.02806
$\sigma_{\beta kin}$
N = 4500 Bandwidth = 0.02485
$\sigma_{\beta rank}$
N = 4500 Bandwidth = 0.02641
$\sigma_{\beta coho}$
N = 4500 Bandwidth = 0.02624
$\sigma_{\beta age}$
N = 4500 Bandwidth = 0.02647
0
1
2
3
4
