# Supplementary material for: Pay-off-biased social learning underlies the diffusion of novel extractive foraging traditions in a wild primate
Source: Proc Biol Sci. 2017 Jun 7;284(1856):20170358. doi: 10.1098/rspb.2017.0358 (PMC5474070; doi:10.1098/rspb.2017.0358)
Supplement: Figure S3 [file rspb20170358supp4.pdf]

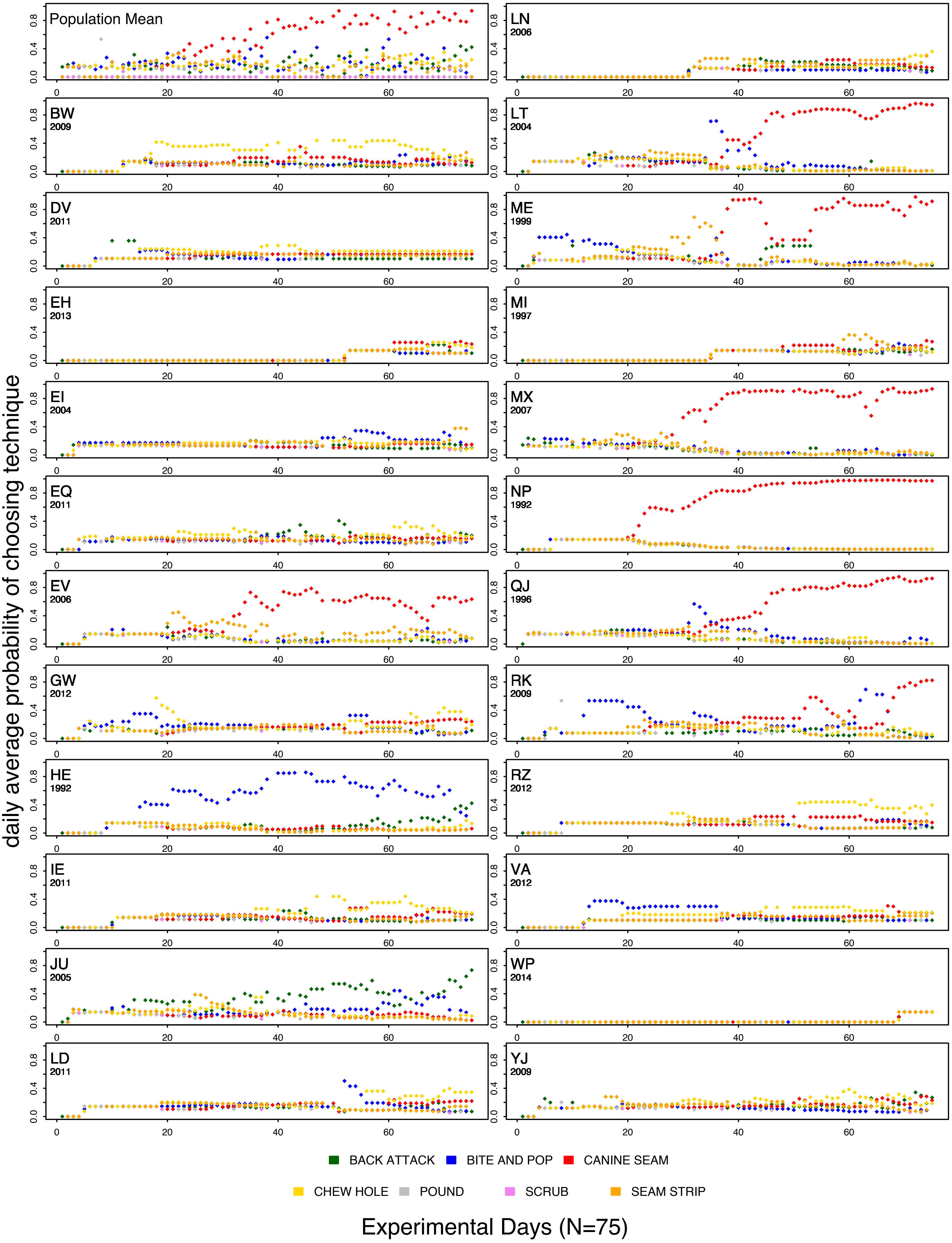
daily average probability of choosing technique
Population Mean
LN 2006
BW 2009
LT 2004
DV 2011
ME 1999
EH 2013
MI 1997
EI 2004
MX 2007
EQ 2011
NP 1992
EV 2006
QJ 1996
GW 2012
RK 2009
HE 1992
RZ 2012
IE 2011
VA 2012
JU 2005
WP 2014
LD 2011
YJ 2009
BACK ATTACK
BITE AND POP
CANINE SEAM
CHEW HOLE
POUND
SCRUB
SEAM STRIP
Experimental Days (N=75)
