# Supplementary figures and images for: Pay-off-biased social learning underlies the diffusion of novel extractive foraging traditions in a wild primate
Source: Proc Biol Sci. 2017 Jun 7;284(1856):20170358. doi: 10.1098/rspb.2017.0358 (PMC5474070; doi:10.1098/rspb.2017.0358)

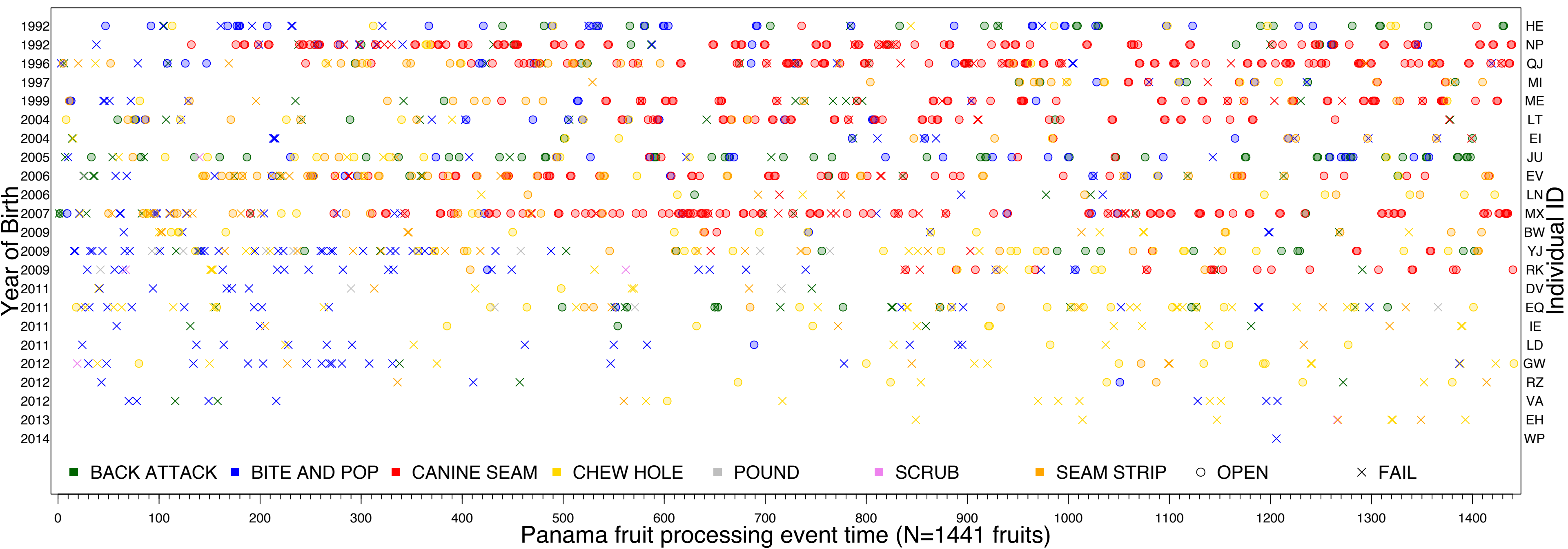

Supplement: Figure S4 [file rspb20170358supp5.pdf]

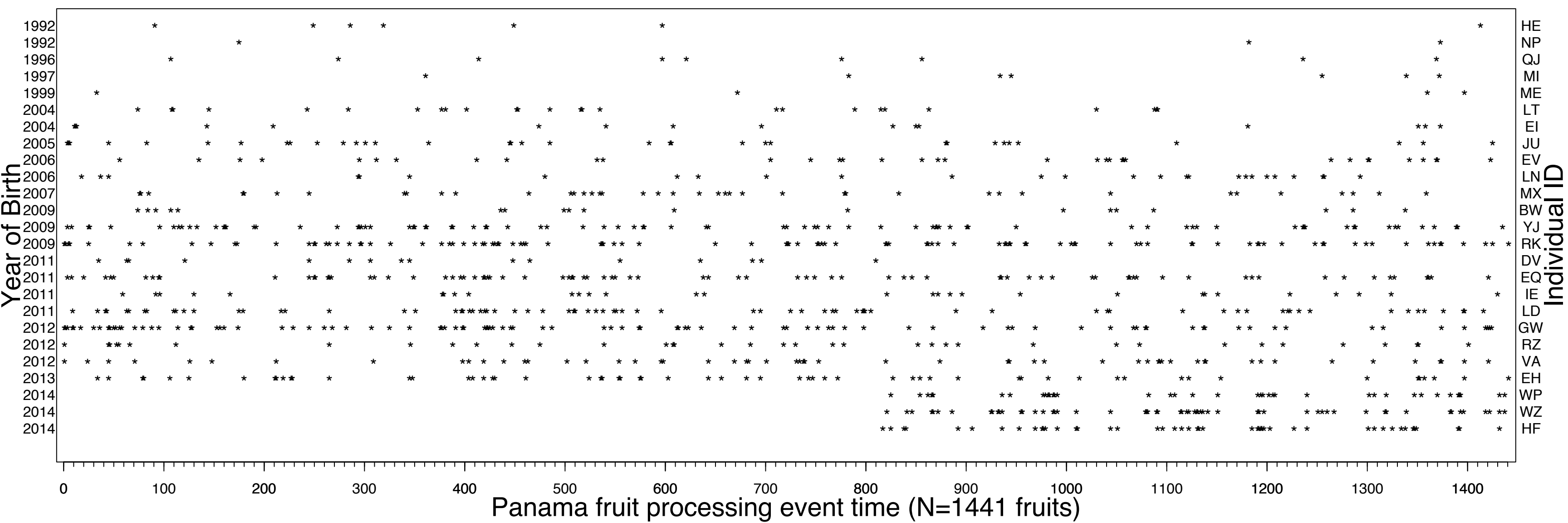

Supplement: Figure S5 [file rspb20170358supp6.pdf]
